# Flug- und Schiffsverkehr während der COVID-19-Pandemie in Deutschland: Herausforderungen für den Öffentlichen Gesundheitsdienst

**DOI:** 10.1007/s00103-021-03297-x

**Published:** 2021-03-17

**Authors:** Scarlett Kleine-Kampmann, Meike Schöll, Lena Ehlers, Elisabeth Hewelt, Udo Götsch, Klaus Göbels, Siegfried Ippisch, Juliane Seidel, Marc Thanheiser, Bert Schindler, Mathias Kalkowski, Matthias Boldt, Martin Dirksen-Fischer, Thomas von Münster, Matthias Jeglitza, Justyna Chmielewska, André Sangs, Barbara Mouchtouri, Ute Rexroth, Maria an der Heiden

**Affiliations:** 1 Institut für Hygiene und Umwelt, Hamburg Port Health Center, Hamburg, Deutschland; 2 Abteilung für Infektionsepidemiologie, Robert Koch-Institut, Berlin, Deutschland; 3 Gesundheitsamt Frankfurt am Main, Frankfurt am Main, Deutschland; 4 Gesundheitsamt Düsseldorf, Düsseldorf, Deutschland; 5 Spezialeinheit – Task Force – Infektiologie und Flughafen, Bayerisches Landesamt für Gesundheit und Lebensmittelsicherheit (LGL), Oberschleißheim, Deutschland; 6 Abteilung für Infektionskrankheiten, Robert Koch-Institut, Berlin, Deutschland; 7 Abteilung Gesundheit, Senatsverwaltung für Gesundheit, Pflege und Gleichstellung, Berlin, Deutschland; 8 Zentralinstitut für Arbeitsmedizin und Maritime Medizin Hamburg, Hamburg, Deutschland; 9 Bundesministerium für Verkehr und Digitale Infrastruktur, Berlin, Deutschland; 10 Bundesministerium für Gesundheit, Berlin, Deutschland; 11 Laboratory of Hygiene and Epidemiology, University of Thessaly (Thessalien), Larisa, Griechenland

**Keywords:** Grenzübergangsstellen, Flughafen, Hafen, Corona, SARS-CoV-2, Points of entry, Airport, Port, Corona, SARS-CoV-2

## Abstract

COVID-19 stellt unsere Gesellschaft seit Januar 2020 vor große Herausforderungen. Durch den globalen Reiseverkehr konnte sich das neue Coronavirus rasch weltweit ausbreiten. In diesem Artikel sollen übersichtsartig die Herausforderungen bei der Implementierung von Maßnahmen im Flug- und Schiffsverkehr aus Sicht des Öffentlichen Gesundheitsdienstes (ÖGD) dargestellt werden. Dies erfolgt anhand einer Auswahl von Ereignissen und Maßnahmen zwischen Januar und August 2020. Die gewonnenen Erkenntnisse werden diskutiert.

Während der COVID-19-Pandemie bewegt sich der ÖGD in einem Spannungsfeld, das sich aus der Dynamik wissenschaftlicher Erkenntnisse, politischer Entscheidungen und der Akzeptanz und Zustimmung in der Gesellschaft ergibt. An Grenzübergangsstellen wie Flug- und Seehäfen gibt es besondere Herausforderungen. Dazu gehören insbesondere Personalengpässe und die Notwendigkeit, Maßnahmen mit hohem organisatorischen Aufwand sehr kurzfristig umzusetzen, wie etwa gesundheitsbehördliche Passagierkontrollen direkt an Luftfahrzeugen, die Einrichtung von Testzentren an Grenzübergangsstellen sowie die Überprüfung der Einhaltung von Quarantänevorgaben. Erschwerend kommt hinzu, dass Passagierlisten, die zur effektiven Kontaktpersonennachverfolgung notwendig sind, oft fehlen oder unvollständig sind. Ebenso fehlen digitale Tools für das Kontaktpersonenmanagement, aber z. B. auch für den Austausch personenbezogener Daten innerhalb des ÖGD. Weitere Schwierigkeiten beim Ausbruchsmanagement ergeben sich durch die engen Verhältnisse an Bord von Schiffen und durch die mögliche psychische Belastung von Besatzung und Passagieren, die in den Maßnahmen noch nicht ausreichend berücksichtigt werden.

Angesichts all dieser Herausforderungen ist es notwendig, den ÖGD langfristig zu stärken – allgemein und an den Grenzübergangsstellen – und den Bund-Länder-Austausch im Flug- und Schiffsverkehr zu intensivieren.

## Abstract (en)

COVID-19 has been challenging our society since January 2020. Due to global travel, the new coronavirus has rapidly spread worldwide. This article aims to provide an overview of the challenges in implementing measures in the air and maritime transport sector from the perspective of the German Public Health Service (Öffentlicher Gesundheitsdienst, ÖGD). Significant events and measures for air and maritime transport between January and August 2020 were selected. Lessons learned are discussed.

During the COVID-19 pandemic, the ÖGD has been operating in a field of tension between the dynamics of scientific knowledge, political decision-making, social acceptance and consent.

There are specific challenges at points of entry such as airports and seaports. These include staff shortages and the need to implement measures with a high organisational effort at very short notice such as health authority passenger checks carried out on aircraft, the establishment of test centres at points of entry and control of compliance with quarantine measures. Aggravating the situation, passenger lists, which are necessary for effective contact tracing, are often not available or incomplete. There is also a lack of digital tools for contact tracing but also, for example, the exchange of personal data within the ÖGD. Further difficulties in outbreak management arise from the cramped conditions on board ships and from the potential psychological stress on crew members and passengers, which have not yet been sufficiently considered.

In view of all these challenges, it is paramount to strengthen the German Public Health Service in general and at points of entry and to intensify the exchange between the national, federal state and local levels.

## Einleitung

Durch den zunehmenden globalen Reiseverkehr können sich Infektionskrankheiten schneller über Landesgrenzen hinweg ausbreiten. Daher richtet sich bei Epi- und Pandemien die mediale und politische Aufmerksamkeit immer wieder auf den internationalen Reiseverkehr und auf Grenzübergangsstellen wie Flughäfen und Häfen. Für die Einschleppung erster Krankheitsfälle spielt der Reiseverkehr bei Pandemien ohne Zweifel eine Rolle. Dennoch sind im weiteren Verlauf des Geschehens die meisten Erkrankungen Folge von Ansteckungen vor Ort. Deshalb ist ein in der Fläche funktionierendes Surveillance- und Gesundheitssystem essenziell in der Bekämpfung und Kontrolle von Infektionskrankheiten.

Die Erwartungen der Politik, der Medien und der Gesellschaft gegenüber den für die Grenzübergangsstellen zuständigen Gesundheitsbehörden sind hoch: Einerseits gehören Reisen für viele beruflich und privat zum Alltag, notwendige Lieferketten (z. B. für Medikamente, persönliche Schutzausrüstung, Lebensmittel) müssen aufrechterhalten werden und der Flug- und Schiffsverkehr soll aufgrund seiner wirtschaftlichen Bedeutung nicht unnötig beeinträchtigt werden. Andererseits soll das Infektionsrisiko für Reisende, für Beschäftigte an den Grenzübergangsstellen und für die Bevölkerung möglichst minimiert werden.

Die Umsetzung des Infektionsschutzes ist angesichts dieser konkurrierenden Erwartungen eine besondere Herausforderung für die zuständigen Gesundheitsbehörden. Zusätzlich unterliegen sowohl die Erkenntnisse zu SARS-CoV‑2 als auch die Maßnahmen während der Pandemie einer großen Dynamik, der der Öffentliche Gesundheitsdienst (ÖGD) gerecht werden soll.

Die COVID-19-Pandemie hat sowohl die Luftfahrtindustrie als auch die maritime Wirtschaft in eine tiefe Krise manövriert. Im Folgenden werden die Herausforderungen des ÖGD im Flug- und Schiffsverkehr während der Pandemie für den Zeitraum Januar bis August 2020 dargestellt und ein Fazit gezogen.

## Rechtliche Rahmenbedingungen

Rechtlich spielen in Deutschland im Bereich der Grenzübergangsstellen neben dem Infektionsschutzgesetz die Internationalen Gesundheitsvorschriften (2005; IGV) und das Gesetz zur Durchführung der Internationalen Gesundheitsvorschriften (IGV-DG; [1–3]) eine besondere Rolle.

**Figure 1:**
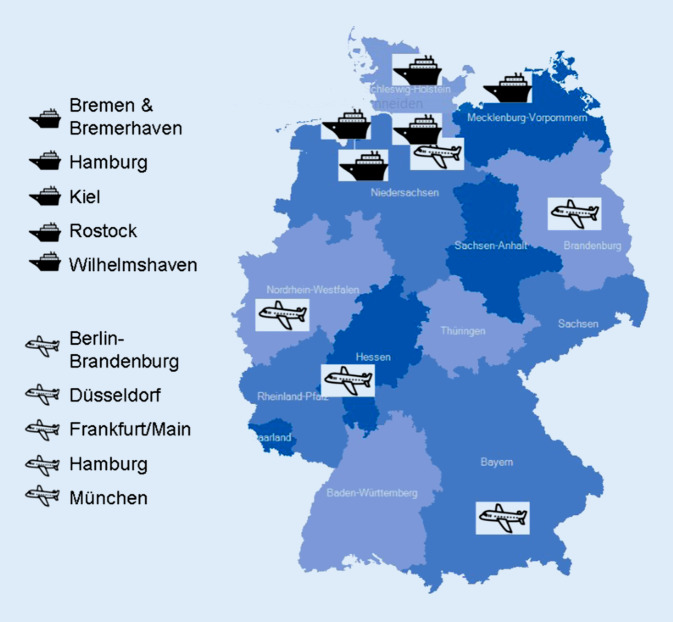

Nach IGV-DG müssen die in Abb. 1 aufgeführten Häfen und Flughäfen Kernkapazitäten für die Überwachung und Reaktion auf Ereignisse vorhalten, die eine gesundheitliche Notlage von internationaler Tragweite darstellen können. Das Robert Koch-Institut (RKI) hat dazu in Zusammenarbeit mit zahlreichen Akteuren und nach Anhörung der obersten Landesgesundheitsbehörden Empfehlungen herausgegeben [4, 5].

## Chronologie ausgewählter Ereignisse im Reiseverkehr

- 30.01.2020: Der Generaldirektor der Weltgesundheitsorganisation (WHO) erklärt die COVID-19-Lage als eine gesundheitliche Notlage internationaler Tragweite (PHEIC; [6])
- Mitte Februar 2020: Einige Fluglinien aus China setzen Flugverkehr aus, Direktflüge aus China nur noch nach Frankfurt am Main und München
- Februar–März 2020: Als Reaktion auf einige schwere Ausbrüche auf Kreuzfahrtschiffen und den daraus folgenden Restriktionen für Häfen anlaufende Schiffe sowie Reisewarnungen wurde die Kreuzschifffahrt weltweit fast vollständig eingestellt.
- 11.03.2020: WHO Generaldirektor stuft Ausbreitung von SARS-CoV‑2 als Pandemie ein [7]
- Mitte März 2020: Weltweit schränken zahlreiche Fluglinien den Flugverkehr ein
- 16. und 17.03.2020: Bundesregierung schließt deutsche Grenzen weitgehend für den Reiseverkehr
- 17.03.2020: Auswärtiges Amt spricht weltweite Reisewarnung aus
- 15.06.2020: Auswärtiges Amt hebt Reisewarnung für die Mitgliedstaaten der Europäischen Union, für Schengen-assoziierte Staaten und das Vereinigte Königreich auf; schrittweise Wiederzunahme des Flugreiseverkehrs [8, 9]
- August 2020: schrittweise Wiederzunahme des Kreuzfahrtbetriebs

## Maßnahmen im Flug- und Schiffsverkehr

Im Zuge der COVID-19-Pandemie wurden zahlreiche Anordnungen seitens des Bundesministeriums für Gesundheit (BMG) als auch der Gesundheitsministerien der Länder mit Relevanz für die Grenzübergangsstellen getroffen [10–15]. Zu den zwischen Januar bis August 2020 angeordneten Maßnahmen zählten u. a. die Verpflichtung zum Ausfüllen von Aussteigekarten (Formulare, in welche einreisende Personen aus definierten Gebieten ihre Erreichbarkeitsdaten und teilweise auch Gesundheitsangaben eintragen müssen), ab April 2020 eine Verpflichtung zur Quarantäne von Einreisenden aus Risikogebieten (Personen mit einer negativen molekularbiologischen SARS-CoV-2-Testung ab Juni 2020 ausgenommen) sowie grenznahe Kontrollen und ein vorübergehendes Landeverbot für Flüge aus der Islamischen Republik Iran.

## Herausforderungen für den ÖGD

### Komplexität der Tätigkeiten an Grenzübergangsstellen

Im Rahmen des internationalen Gesundheitsschutzes ist es Aufgabe v. a. der für die Grenzübergangsstellen zuständigen Gesundheitsbehörden, Reisende umfassend über mögliche Infektionsrisiken zu informieren und Infektionsketten zügig zu unterbrechen. Dies erfordert eine enge Kooperation mit Beförderern im grenzüberschreitenden Flug- oder Schiffsverkehr sowie mit Betreibern von Flughäfen und Häfen.

**Figure 2:**
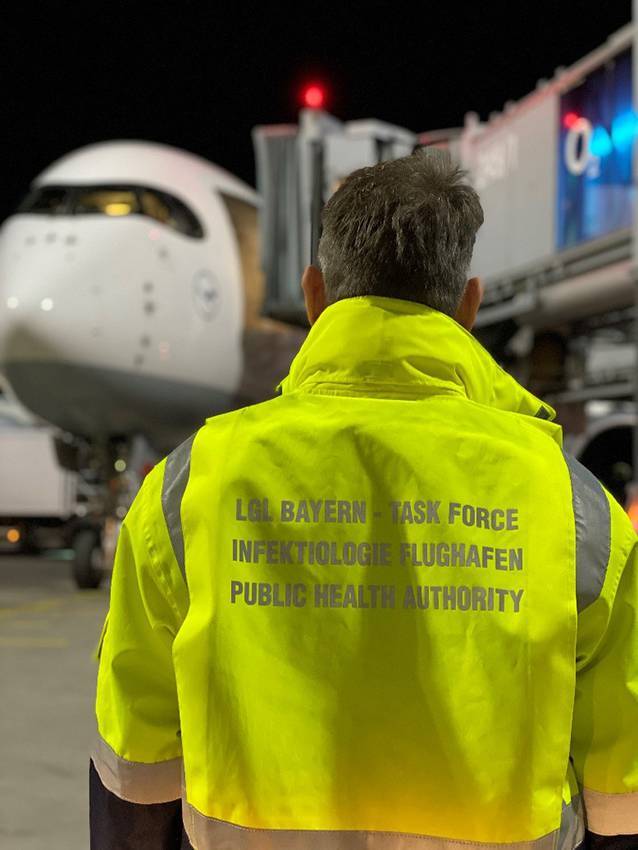

**Figure 3:**
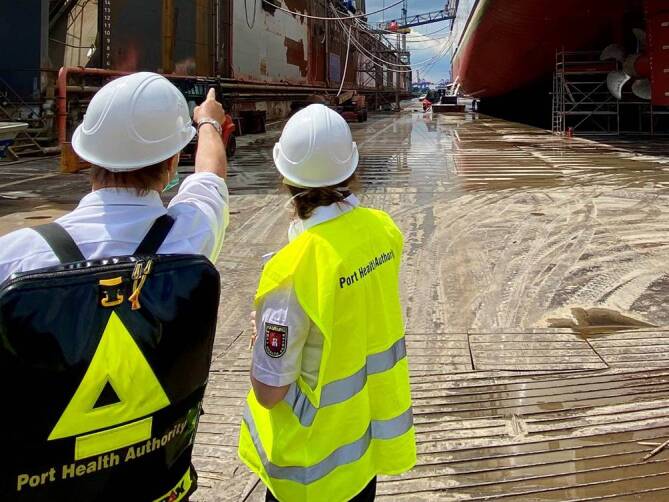

Zusätzliche Besonderheiten für die Tätigkeit des ÖGD an den Grenzübergangsstellen:

- Heterogenität der Flug‑, See- und Binnenhäfen hinsichtlich Passagieraufkommen, Herkunft der Reisenden, Anzahl der beteiligten Beförderer sowie Saisonalität (Kreuzfahrtsaison i. d. R. von April bis Dezember)
- Heterogenität der für die Flughäfen und Häfen zuständigen Gesundheitsbehörden: Während beispielsweise für den Flughafen Düsseldorf nur das örtliche Gesundheitsamt zuständig ist, ist für den Flughafen und Hafen Hamburg das Hamburg Port Health Center (HPHC) als zusätzliche Behörde zuständig und für den Flughafen München zusätzlich zu dem örtlichen Gesundheitsamt Erding die „Spezialeinheit – Task Force – Infektiologie und Flughafen“ des Bayerischen Landesamtes für Gesundheit und Lebensmittelsicherheit (LGL; Abb. 2). Die Flughäfen Frankfurt am Main und München verfügen als Besonderheit über einen medizinischen Dienst vor Ort, 24 h, 7 Tage die Woche
- Besonderheiten im Arbeitsalltag bezüglich der Arbeitssicherheit (z. B. regelmäßige Schulungen zum Verhalten auf einem Hafenterminal oder dem Vorfeld), der Akteure (z. B. Flughafenfeuerwehr in Hamburg versus allgemeine Feuerwehr in anderen Flughäfen), der z. T. langen Anfahrtswege (von der Gesundheitsbehörde zum Flughafen oder zu bestimmten Hafenterminals oder Anlegestellen) und der Sicherheitsvorkehrungen und -kontrollen (Abb. 3)

Die Tätigkeit des ÖGD an den unterschiedlichen Grenzübergangsstellen erfordert besondere Kenntnisse, Netzwerke und Ressourcen. Die Pandemiebewältigung an den Grenzübergangsstellen von Januar bis August 2020 gelang nur unter beispiellosem Einsatz der wenigen vorhandenen Mitarbeiterinnen und Mitarbeiter sowie kurzfristiger Einbindung zahlreicher zusätzlicher Kräfte. Eine nachhaltige personelle und finanzielle Stärkung des ÖGD an allen Grenzübergangsstellen, inklusive der nicht IGV-benannten Flughäfen und Häfen, ist zwingend notwendig.

### Informationsaustausch zwischen den Akteuren des ÖGD

Um der Dynamik der Lage und der Umsetzung umfangreicher Maßnahmen gerecht zu werden, war ein regelmäßiger Informationsaustausch zwischen Akteuren des ÖGD essenziell. Im Bereich „Flugverkehr“ existiert hierfür bis dato kein formelles Bund-Länder-Gremium im ÖGD, seit Beginn der Pandemie tauscht sich jedoch regelmäßig eine informelle Arbeitsgemeinschaft IGV-benannter Flughäfen aus. Im Rahmen eines Verwaltungsabkommens der norddeutschen Bundesländer über die Zusammenarbeit auf dem Gebiet der Schifffahrtsmedizin wurde 1974 formell der Arbeitskreis der Küstenländer (AkKü) etabliert, bei dem das BMG, das Bundesministerium für Verkehr und digitale Infrastruktur (BMVI) und das RKI Gaststatus haben. Die Voraussetzungen, ein solches Abkommen samt flankierender Kooperationsvereinbarungen auf den Flugverkehr zu übertragen, sind nicht ohne Weiteres gegeben.

### Verwendung und Anpassung von Aussteigekarten

Im Flugverkehr wird seit Jahren eine international durch die WHO und die Internationale Zivilluftfahrtorganisation (ICAO) standardisierte Aussteigekarte verwendet, die bei Verdacht auf eine übertragbare Krankheit an Bord an ansteckungsverdächtige Personen (Kontaktpersonen) zur Erhebung von Erreichbarkeitsdaten ausgeteilt werden soll, um ggf. über weitere Maßnahmen informieren zu können [16].

Im Laufe der COVID-19-Pandemie wurde diese Aussteigekarte für verschiedene Zwecke angepasst: Nach Anordnung des BMG vom 29.01.2020 musste diese von Reisenden, die von einem Flughafen in der Volksrepublik China gestartet waren, ausgefüllt werden [14]. In verschiedenen Anordnungen erfolgten Ausdehnungen dieser Pflicht auf die zum jeweiligen Zeitpunkt ausgewiesenen Risikogebiete.

Im Februar 2020 wurden zeitweise auch Gesundheitsfragen aufgenommen („Aussteigekarten mit Selbstauskunft“). Dies wurde kontrovers diskutiert, da Zweifel an der Wahrheitstreue bei der Beantwortung bestanden. Herausforderungen ergaben sich für die Behörden bei der Kontrolle der Antworten, der organisatorischen Umsetzung (z. B. Umgang mit Transitreisenden) und bei der erforderlichen Compliance der Reisenden. Ab 10.03.2020 wurden die Gesundheitsfragen gestrichen, nachdem beispielsweise in Frankfurt am Main kein einziger Fall von COVID-19 durch das Verfahren entdeckt worden war.

Mit der Anordnung des BMG vom 06.08.2020 wurden die Aussteigekarten erneut angepasst [10]. Sie sollten den Gesundheitsbehörden in Deutschland zur Verfügung gestellt werden, um die Einhaltung der länderspezifischen Quarantäneverordnungen im Zusammenhang mit Einreise aus Risikogebieten stichprobenartig zu überprüfen. Herausforderungen lagen in der Praktikabilität der papierbasierten Aussteigekarten, in der häufig unzureichenden Qualität und Vollständigkeit der Angaben und der geringen Akzeptanz. Zudem sind abweichende nationale Lösungen im internationalen Reiseverkehr nur von begrenztem Mehrwehrt. Die papierbasierte Aussteigekarte wurde im Herbst 2020 durch eine digitale Einreiseanmeldung abgelöst.

### Effiziente Kontaktpersonennachverfolgung

Die Kontaktpersonennachverfolgung nach Exposition in einem Transportmittel stellt den ÖGD vor zahlreiche Herausforderungen, beginnend mit der Frage nach der zuständigen Ansprechperson bei den Beförderern bis hin zum Erlangen einer vollständigen Passagierliste in angemessener Zeit, mit der die Kontaktpersonen erreicht werden können [17].

Um eine umfassende und zeitnahe Kontaktpersonenermittlung im Transportbereich zu ermöglichen, sind 2 technische Alternativen denkbar: 1.) Verbesserung der Qualität, Vollständigkeit und Freigabe der Passagierdaten, die durch die Beförderer erhoben werden, oder 2.) Vorabregistrierung von Reisenden im grenzüberschreitenden Verkehr, wie es in anderen Ländern z. T. bereits umgesetzt ist. In beiden Fällen wäre eine EU-weit einheitliche digitale Lösung zur Kontaktpersonennachverfolgung anzustreben, um den ÖGD zu entlasten.

### Dynamische Ausweisung von Risikogebieten

Zwischen Januar und April 2020 wies das RKI Risikogebiete aus, in denen u. a. von anhaltender Mensch-zu-Mensch-Übertragung ausgegangen wurde. Seit dem 10.04.2020 hat das RKI auf eine Ausweisung von internationalen Risikogebieten wegen der zu diesem Zeitpunkt bereits weltweiten Verbreitung von SARS-CoV‑2 verzichtet. Stattdessen traten ab Juni 2020 Quarantäneverordnungen der Bundesländer in Kraft, die auf Risikogebiete Bezug nahmen, die vorab durch das BMG, das Auswärtige Amt und das Bundesministerium des Innern, für Bau und Heimat (BMI) festgelegt worden waren. Diese neu bewerteten Risikogebiete ersetzten die des RKI, wurden aber weiterhin durch das RKI veröffentlicht [18]. Einreisende aus diesen Gebieten unterlagen grundsätzlich einer Quarantänepflicht nach Maßgabe der Bundesländer. An die Ausweisung von Risikogebieten wurden verschiedene Verordnungen und Anordnungen gekoppelt (z. B. die Ausgabe von Aussteigekarten), was aufgrund der Dynamik des Geschehens sowohl Behörden als auch Beförderer vor große Herausforderungen stellte. Insbesondere erwies es sich für den ÖGD als schwierig, alle diesbezüglich notwendigen Maßnahmen sofort umzusetzen. Ein Vorlauf von mindestens 24 h bis Inkrafttreten der Maßnahmen ist generell für den ÖGD dringend notwendig, um z. B. vorzuhaltende Test- und Personalkapazitäten auszubauen.

### Rückholaktionen für deutsche Staatsangehörige und ihre Familien

Im März 2020 startete das Auswärtige Amt eine beispiellose Rückholaktion v. a. für deutsche Staatsangehörige und ihre Familien aus dem Ausland. Insgesamt wurden 236.000 Reisende zurückgeholt, vorwiegend per Flugzeug. Das Auswärtige Amt organisierte 260 Sonderflüge, überwiegend mit den Zielflughäfen Frankfurt am Main und München. Um eine gleichmäßige Verteilung der Repatriierungsflüge an Flughäfen in Deutschland wurde gebeten, dies war aber zumeist nicht möglich.

### Befreiung von Quarantänevorgaben durch PCR-Tests

Mit der von Bund und Ländern beschlossenen Quarantäne-Muster-Verordnung vom 08.04.2020 wurde die Grundlage für eine verpflichtende 14-tägige Quarantäne nach Einreise aus Risikogebieten geschaffen [19]. Die Einhaltung der Quarantäne konnte von den Behörden aber allenfalls stichprobenartig geprüft werden. Die Befreiung von der Quarantäne durch einen negativen PCR-Test auf SARS-CoV‑2 unmittelbar nach Einreise wurde von der Fachebene in Bund und Ländern kritisch gesehen, da Personen, die sich zum Zeitpunkt der Testung in der Inkubationsphase befinden, hierdurch nicht erfasst werden. Ab August 2020 hatten Reiserückkehrende Anspruch auf eine kostenlose PCR-Testung (nachträgliche Ergänzung: Ab Dezember 2020 mussten die Kosten von den Reisenden selbst getragen werden). Innerhalb kurzer Zeit waren an allen operativen IGV-benannten Flughäfen Möglichkeiten zur Testung von Reisenden etabliert.

### Umgang mit Krankheitsverdachtsfällen auf Schiffen

Schiffe inklusive ihrer Besatzung befanden sich pandemiebedingt für Monate in quarantäneähnlichen Zuständen. Landgang war den meisten Besatzungsmitgliedern aufgrund von Einreiseregelungen untersagt. Die Mehrheit der Reedereien sahen strenge Kontaktbeschränkungen und Hygienemaßnahmen vor. Trotz oder gerade aufgrund dieser Maßnahmen kam es immer wieder zu unentdeckten Infektionsketten an Bord von Schiffen.

Das Erkennen von einzelnen Krankheitsfällen oder Ausbrüchen an Bord von Schiffen wurde durch einen hohen Anteil von oligo- und asymptomatischen Infizierten und eingeschränkter Diagnostik an Bord erschwert [20, 21]. Die Erfahrungen der Hafenärztlichen Dienste (HÄD) zeigen auch, dass Passagiere und Besatzungsmitglieder aus verschiedenen Gründen bei der Meldung von Erkrankungsanzeichen und der Inanspruchnahme ärztlicher Hilfe zurückhaltend sein können. Die Organisationsstruktur an Bord ist meist durch strenge Hierarchien charakterisiert. Auch die Schiffsärztin oder der Schiffsarzt befindet sich im Offiziersrang. Ansonsten wird die medizinische Betreuung meist an den zweiten nautischen Offizier delegiert. Krankheitsfälle unter Besatzungsmitgliedern werden somit meistens dem direkten Vorgesetzten kommuniziert, was die erkrankte Person als Arbeitnehmer in eine prekäre Lage bringen kann [22]. Für Passagiere können Krankmeldungen und Quarantänemaßnahmen zu unkalkulierbaren Kosten und Einschränkungen der Weiterreise führen. Kreuzfahrtunternehmen stehen durch die COVID-19-Pandemie unter einem hohen ökonomischen Druck und müssen erhebliche Imageschäden befürchten [23]. Zudem besteht die Befürchtung, dass einem Schiff aufgrund von Krankheits- oder Verdachtsfällen an Bord das Einlaufen in einem Hafen nicht gewährt wird [24].

### Ausbruchsmanagement an Bord eines Schiffes

Im August 2020 wurden auf einem norwegischen Expeditionskreuzfahrtschiff 4 Besatzungsmitglieder positiv auf SARS-CoV‑2 getestet. Die Behörden hatten Schwierigkeiten einen Großteil der bereits von Bord gegangenen Gäste zu kontaktieren [25]. Das Beispiel unterstreicht, dass die Absonderung ansteckungs- und krankheitsverdächtiger Personen die zentrale Schutzmaßnahme darstellt und in begründeten Verdachtsfällen unabhängig von Testverfügbarkeit und -ergebnissen erfolgen muss.

Bestätigt sich ein Verdachtsfall auf einem Schiff, wird durch den HÄD eine Ausbruchsuntersuchung an Bord durchgeführt. Diese beinhaltet neben der Identifizierung von Kontaktpersonen auch eine Schiffshygienekontrolle, um mögliche Infektionsquellen und Übertragungswege zu detektieren. Insbesondere Kabinen- und Tischnachbarn, befreundete Besatzungsmitglieder, Personen der gleichen Wachgruppe und Passagiere, die an gemeinsamen Aktivitäten teilgenommen haben, werden in der Regel als enge Kontaktperson (Kategorie 1 – erhöhtes Infektionsrisiko) eingestuft. Kontaktpersonen der Kategorie 2 (geringeres Infektionsrisiko) sind aufgrund der räumlichen Enge an Bord eines Schiffes schwer abzugrenzen [26]. Die Einstufung der Kontaktpersonen stellt einen zentralen Punkt der behördlichen Maßnahmen dar und muss unter dem juristischen Aspekt des Freiheitsentzugs sorgsam abgewogen und begründet werden.

Schiffe sind für die Umsetzung von Isolations- und Quarantänemaßnahmen meist nicht gut geeignet, da enge Verhältnisse vorherrschen und oft unklar ist, ob die raumlufttechnischen Anlagen ausreichend gut funktionieren. Eine frühzeitige Evakuierung stellt die effektivste Maßnahme dar, die Infektionskette zu durchbrechen [20]. Auch im Hinblick auf die Behandlung von komplizierten Verläufen und die psychosoziale Betreuung sollte zeitnah eine Unterbringung in geeigneten Einrichtungen an Land erfolgen. Die Länder sind verpflichtet, Räume und Einrichtungen zur Durchführung von Absonderungsmaßnahmen vorzuhalten (§ 30 Absatz 7 IfSG; [2]). Darüber hinaus bestehen gemäß § 13 Absatz 4 und 5 IGV-DG [1] und Empfehlungen des RKI Anforderungen an Kernkapazitäten der IGV-benannten Häfen [4]. Die Praxis zeigt, dass die Bereitstellung geeigneter Quarantäneeinrichtungen auch in großen IGV-benannten Häfen eine Herausforderung darstellt. Kreuzfahrtschiffe müssen ebenfalls Planungen für eine sichere Isolierung und Quarantäne an Bord treffen und bei der Belegung der Kabinen die Kapazitäten berücksichtigen.

Bei der Anordnung einer Quarantäne für Besatzungsmitglieder eines Schiffes ist zu berücksichtigen, dass ein Schiff auch im Hafen liegend mit einer definierten Minimalbesatzung ausgestattet sein muss, um die Schiffssicherheit zu gewährleisten.

Die Anordnung und Überwachung der Maßnahmen bei einem Ausbruchsgeschehen inkl. Untersuchungen, Befragungen, Beratung und Kommunikation mit allen beteiligten Akteuren sind sehr zeit- und ressourcenintensiv und bedeuten einen erhöhten Koordinierungsbedarf durch den HÄD.

### Psychische Belastung der Betroffenen an Bord

Die Beschränkung von Grundrechten durch Absonderungsmaßnahmen kann zu einer erheblichen Verunsicherung und Belastung der Betroffenen sowie zu existenziellen Sorgen der Besatzungsmitglieder führen. Unvollständige Informationen und Kommunikationsbarrieren können die Verunsicherung und das Gefühl des Ausgeliefertseins verstärken, Konfliktsituationen können entstehen. Eine enge psychosoziale Unterstützung der Betroffenen ist dringend geboten. Sorgen und Ängste müssen wahrgenommen und auf individuelle Bedürfnisse eingegangen werden. Der HÄD arbeitet im engen Austausch mit Mitarbeitenden der Deutschen Seemannsmission. Das Havariekommando, eine gemeinsame Einrichtung des Bundes und der Küstenländer, kann in Bedarfsfällen eine psychosoziale Notfallversorgung zur Unterstützung an Bord koordinieren.

### Anforderungen zur Wiederzunahme des Flugverkehrs

Nach dem Einbruch des Flugverkehrs Mitte März 2020 wurden im Mai 2020 angesichts fallender Erkrankungszahlen die Bedingungen für eine Wiederzunahme diskutiert. Von der European Aviation Safety Authority (EASA) und dem European Centre for Disease Prevention and Control (ECDC) wurden dazu seit dem 21.05.2020 Empfehlungen veröffentlicht, die beispielsweise das Tragen von Masken am Flughafen und im Luftfahrzeug vorsahen [27]. Daraufhin wurden „Hinweise für COVID-19-Prozesse im Flugverkehr“ [28] veröffentlicht, um in Deutschland eine Orientierungshilfe für Maßnahmen und Prozesse im Luftfahrzeug und an Flughäfen zu geben. Sie beinhalteten u. a. konkrete Hinweise zum Tragen von Mund-Nasen-Bedeckung, zur Einhaltung der Abstandsregeln und zur Lüftung sowohl im Flughafenbereich als auch während des Aufenthalts im Luftfahrzeug. Ab dem 15.06.2020 nahm der Flugverkehr unter Berücksichtigung entsprechender Hygienekonzepte wieder zu (Stand September 2020).

### Anforderungen zur Wiederaufnahme des Kreuzfahrtbetriebes

Im Juni 2020 fanden Treffen zur Vorbereitung auf eine Wiederaufnahme des Kreuzfahrtbetriebes in Hamburg statt. Vertreten waren der Dachverband führender internationaler Kreuzfahrtverbände, die Hafenbehörde, der HÄD, die Wasserschutzpolizei, die Terminalbetreiber und die Behörde für Wirtschaft, Verkehr und Innovation. Unter Beteiligung des AkKü und der Hafenkapitäne der deutschen Seehäfen erstellten sie Leitsätze zur Wiederaufnahme des Kreuzfahrtbetriebes. Danach haben Kreuzfahrtverbände vor Reisebeginn für jedes Schiff individuelle Konzepte vorzulegen, in denen Melde- und Kommunikationswege, Pläne für die Durchführung von PCR-Testungen, die Umsetzung von Quarantänemaßnahmen an Bord, die maximale Passagierzahl und Kabinenbelegung sowie weitere Maßnahmen zur Vermeidung der Ausbreitung von SARS-CoV‑2 dargestellt werden.

Unter Voraussetzung der Einhaltung der vereinbarten Leitsätze und der geltenden länderspezifischen Verordnungen wurde den Kreuzfahrtverbänden zugesichert, dass die norddeutschen Häfen für den Anlauf der Schiffe und die Notfallversorgung gemäß IGV offen bleiben. Die Unternehmen agierten bei der Wiederaufnahme unterschiedlich, der Kreuzfahrtbetrieb lief schrittweise und unter großen Einschränkungen wieder an.

## Gewonnene Erkenntnisse

Die COVID-19-Pandemie hat den Reiseverkehr weitgehend verändert. Die Komplexität der Aufgaben, die Durchmischung von Personen in beengten Räumlichkeiten und die hohe Dynamik von Passagierströmen stellen den ÖGD an Grenzübergangsstellen vor besondere Herausforderungen. Die Maßnahmen des ÖGD müssen verständlich und nachvollziehbar mit allen beteiligten Akteuren kommuniziert werden, um Akzeptanz zu schaffen. Die Akzeptanz kann beeinträchtigt werden, wenn Maßnahmen inkongruent zu fachlichen Einschätzungen angeordnet werden (z. B. frühzeitige Befreiung von einer Quarantäneverfügung durch Testung).

Die Vernetzung mit den beteiligten Akteuren national und international ist für die Arbeit des ÖGD an Grenzübergangsstellen essenziell. Zusätzliches Personal muss teils kurzfristig und auch nachts sowie an Sonn- und Feiertagen zur Verfügung stehen. Etablierte (Notfall‑)Pläne und Instrumente (z. B. Aussteigekarten) müssen vor den gesetzlichen Rahmenbedingungen stetig angepasst werden. Der ÖGD ist auch hier als Vermittler und Beteiligter zwischen der Praxis, Politik und der Öffentlichkeit gefordert. Damit der ÖGD den Anforderungen des Infektionsschutzes im internationalen Reiseverkehr gerecht werden kann, sollten daher folgende Voraussetzungen erfüllt werden:

### 1. Allgemeine, nachhaltige Stärkung des ÖGD.

Meldungen zu SARS-CoV-2-Infektionen bei Reisenden erfolgen bei dem für den Wohn- oder Aufenthaltsort zuständigen Gesundheitsamt, welches auch die entsprechenden Maßnahmen, wie etwa die Ermittlung von Kontaktpersonen zur Unterbrechung von Infektionsketten, einleitet. Zur Bekämpfung und Kontrolle von Infektionskrankheiten bei Reisenden ist somit ein in der Fläche funktionierender ÖGD mit speziell ausgebildetem Fachpersonal essenziell. Entsprechende Aus- und Fortbildungsmöglichkeiten müssen gestärkt werden.

### 2. Nachhaltige Stärkung des ÖGD spezifisch an Grenzübergangsstellen.

**Figure 4:**
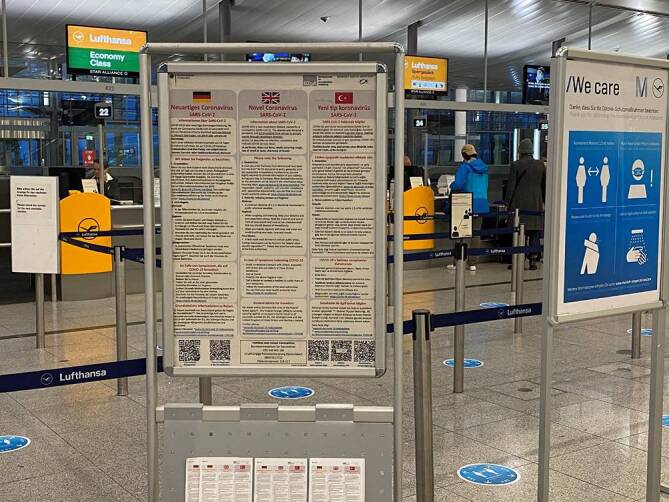

An Grenzübergangsstellen sollten personelle und finanzielle Kapazitätsengpässe im ÖGD beseitigt werden, damit dieser im Bedarfsfall kurzfristig 24/7 vor Ort tätig werden kann und dabei den vielfältigen Aufgaben gerecht wird. Zu den Aufgaben zählen: Einsatzführung bei biologischen Ereignissen, gezielte Aufklärung und Information an Reisende (Abb. 4), Durchführung von Hygienekontrollen in Transportmitteln und Umsetzung sämtlicher weiterer Infektionsschutzmaßnahmen. Nach § 8 Absatz 5 und § 13 Absatz 5 IGV-DG müssen Notfallpläne vom Hafenbetreiber und Flughafenunternehmer geschaffen und unterhalten werden. Der ÖGD an Grenzübergangsstellen hat eine koordinierende und überwachende Funktion, bei der er sich mit den vielfältigen Akteuren abstimmen muss. Dazu zählt auch die Einbindung psychosozialer Dienste, insbesondere im Schiffsverkehr. Die COVID-19-Lage hat gezeigt, dass die Personalkapazitäten in einer Pandemie sehr kurzfristig (z. B. über Amtshilfe) und umfangreich ausgebaut werden müssen. Zur Unterstützung der Maßnahmen an Grenzübergangsstellen muss zusätzliches Personal adäquat eingeführt, geschult und begleitet werden. In Nichtkrisenzeiten sollten die Pläne regelmäßig beübt werden, um ggf. Schwachstellen zu finden und Prozesse anzupassen.

### 3. Stärkung und Verzahnung der Netzwerke und Gremien für Grenzübergangsstellen.

Um in Krisensituationen wie der COVID-19-Pandemie handlungsfähig zu sein, sollten Netzwerke und Gremien, die für Grenzübergangsstellen zuständig sind, dauerhaft gestärkt und untereinander sinnvoll verzahnt werden. Ziele sind: die Entwicklung von harmonisierten Handlungsleitfäden und praxisnahen Maßnahmen, Schulungs- und Übungsmaterialien, der Erfahrungsaustausch zum Umgang mit Maßnahmen sowie die evidenzbasierte Beratung politischer Entscheidungsgremien. Politische Entscheidungen sollten mit einer ausreichenden Vorlaufzeit an die für die Umsetzung verantwortlichen Behörden kommuniziert werden.

### 4. Implementierung und Nutzung digitaler Systeme.

Im ÖGD ist eine zeitgemäße, datenschutzkonforme Implementierung und Nutzung digitaler Systeme notwendig, z. B. für das Kontaktpersonenmanagement im Transportsektor, für den Umgang mit Aussteigekarten und ganz allgemein zum Austausch personenbezogener Daten im ÖGD zwischen allen relevanten Behörden. Für die Nutzung digitaler Systeme beim Kontaktpersonenmanagement im Transportsektor sprachen sich auch die Chief Medical Officers im Rahmen der deutschen EU-Ratspräsidentschaft am 29.09.2020 aus [29].

### 5. Einbeziehung aller Flughäfen und Häfen mit internationalem Reiseverkehr.

Durch die COVID-19-Pandemie wurde deutlich, dass das Konzept der IGV-benannten Flughäfen und Häfen zu kurz gegriffen ist, da im Pandemiefall rasch alle Grenzübergangsstellen betroffen sein können. Ein Konzept zur Vernetzung des ÖGD an allen Flughäfen und Häfen mit internationalem Reiseverkehr sollte erstellt werden; alle Grenzübergangsstellen mit relevantem internationalen Reiseverkehr sollten bestimmte Kernkapazitäten vorhalten (z. B. Notfallpläne für den Umgang mit krankheits- und ansteckungsverdächtigen Personen). Dadurch könnten z. B. auch Rückholaktionen für deutsche Staatsangehörige aus dem Ausland auf breitere Schultern verteilt werden.

### 6. Berücksichtigung von Ausnahmeregelungen bei Einreise und Quarantäne für Besatzungen.

Die Ausnahmeregelungen der Einreise- und Quarantänebestimmungen sollten Besatzungen im Flug- und Schiffsverkehr (auch aus Drittländern) möglichst bundesweit einheitlich berücksichtigen.

### 7. Verpflichtung zur Benennung von Kontaktstellen für den ÖGD und Einstufung des ÖGD als Sicherheitsbehörde.

Um eine effiziente Zusammenarbeit zwischen dem ÖGD, den Betreibern von Flug- und Schiffshäfen sowie den Beförderern zu ermöglichen, sollten feste und erreichbare Anlaufstellen etabliert sein. Die Gesundheitsbehörde mit Zuständigkeit für IGV-Grenzübergangsstellen ist in sehr sensible und zeitkritische Einsatz- und Notfallplanungen involviert. Sie muss im Einsatz- und Ereignisfall ähnlich wie Polizei oder Notversorgung schnellstmöglich vor Ort sein, um schwere gesundheitliche Schäden bei Einsatzkräften, Beschäftigten und der Bevölkerung oder Gefahren für die öffentliche Sicherheit oder Ordnung abzuwenden oder bedeutende Sachwerte zu erhalten. Für diese Aufgaben ist, wo noch nicht erfolgt, eine Einstufung des ÖGD als Sicherheitsbehörde (Behörde und Organisation mit Sicherheitsaufgaben – BOS) mit unverzüglichem Zutritt zum Sicherheitsbereich, Behördenfunk sowie Sonder- und Wegerechten unabdingbar.

## Fazit

Der ÖGD ist während der COVID-19-Pandemie im Flug- und Schiffsverkehr vor zahlreiche Herausforderungen gestellt worden. Der am 05.09.2020 beschlossene „Pakt für den ÖGD“ ist ein wichtiger Schritt zur Stärkung des ÖGD, insbesondere auch an Grenzübergangsstellen [30]. Die Berücksichtigung der in diesem Artikel genannten Lösungsvorschläge bei der Implementierung des Pakts ist aus Sicht der Autorinnen und Autoren dringend empfohlen.
